# Optimizing the two-step floating catchment area method for measuring spatial accessibility to medical clinics in Montreal

**DOI:** 10.1186/1472-6963-11-166

**Published:** 2011-07-11

**Authors:** André Ngamini Ngui, Philippe Apparicio

**Affiliations:** 1 Douglas Mental Health University Institute, 6875 Bld. Lasalle, Verdun, Montréal (Québec), H4H 1R3, Canada; 2 Spatial Analysis and Regional Economics Laboratory, Université du Québec, Institut national de la recherche scientifique, Centre Urbanisation, Culture Société, 385 rue Sherbrooke est, Montréal (Québec), H2X 1E3, Canada

**Keywords:** Spatial accessibility, medical clinics, health services, optimized two step-floating catchment area, Montreal

## Abstract

**Background:**

Reducing spatial access disparities to healthcare services is a growing priority for healthcare planners especially among developed countries with aging populations. There is thus a pressing need to determine which populations do not enjoy access to healthcare, yet efforts to quantify such disparities in spatial accessibility have been hampered by a lack of satisfactory measurements and methods. This study compares an optimised and the conventional version of the two-step floating catchment area (2SFCA) method to assess spatial accessibility to medical clinics in Montreal.

**Methods:**

We first computed catchments around existing medical clinics of Montreal Island based on the shortest network distance. Population nested in dissemination areas were used to determine potential users of a given medical clinic. To optimize the method, medical clinics (supply) were weighted by the number of physicians working in each clinic, while the previous year's medical clinic users were computed by ten years age group was used as weighting coefficient for potential users of each medical clinic (demand).

**Results:**

The spatial accessibility score (SA) increased considerably with the optimisation method. Within a distance of 1 Km, for instance, the maximum clinic accessible for 1,000 persons is 2.4 when the conventional method is used, compared with 27.7 for the optimized method. The t-test indicates a significant difference between the conventional and the optimized 2SFCA methods. Also, results of the differences between the two methods reveal a clustering of residuals when distance increases. In other words, a low threshold would be associated with a lack of precision.

**Conclusion:**

Results of this study suggest that a greater effort must be made ameliorate spatial accessibility to medical clinics in Montreal. To ensure that health resources are allocated in the interest of the population, health planners and the government should consider a strategy in the sitting of future clinics which would provide spatial access to the greatest number of people.

## Background

Accessibility to medical clinics is a contentious issue both in the third world [1-3] and in developed countries [4-6]. Poor access to medical clinics may result in people with simple health problems not consulting a health professional and subsequently developing more complex conditions with irreversible consequences [7]. The Canada Health Act (CHA) recognizes the importance of access to healthcare and states that all Canadians are entitled to receive medical services without barriers or restrictions. At the same time, Canadian provincial health systems are obligated to ensure access to health services for all citizens, based primarily on the principles of "universality" and "accessibility" which is enshrined in the Canada Health Act adopted in 1984 [8].

Given these guidelines aimed at reducing barriers in access to health care, it is important to note that recent studies still report disparities in access to healthcare in Canada [4,9-11]. In Canada, as in many other developed countries, the geographic distribution of physicians does not necessarily match that of population since access to healthcare is affected by where physicians locate (supply) and where people reside (demand). However, interpreting this distribution is difficult due to the multiple definitions of "access" and the lack of specifications on how access should be measured [5,12].

Accessibility to healthcare entails a complex set of factors and processes including service providers, transportation networks (for instance, travel time), individual socioeconomic characteristics, each decision-making strategies, and consumer's ability to pay for services[13]. Accessibility may also be defined in terms of affordability, acceptability, availability and spatial accessibility [12]. Although it is multidimensional, accessibility can be grouped in four main categories: potential or revealed, spatial or aspatial [14,15].

Potential accessibility focuses on the probable utilization of services, given the population size and its demographics, while revealed accessibility concerns actual use of services. Spatial access analyses the importance of spatial separation between supply and demand as a barrier or a facilitator and aspatial access focuses on nongeographic barriers or facilitators [14]. Our study focuses on potential spatial accessibility because we seek to assess probable accessibility to medical clinics by the population.

Previous studies carried out by economists and epidemiologists focused on revealed aspatial access to healthcare [16-18]. More recently, the increasing availability of geographical information systems (GIS) together with the proliferation of spatially disaggregate data has led to an improved analytical and evidence base with which to identify and target those groups and areas with poorer accessibility and physician shortage [19,20]. GIS measures range from counting the number of services contained within census tract boundaries [21] to reporting the number of facilities inside a given Euclidean or Travel-Time distance of demand points [5,22].

A number of studies have used GIS when studying accessibility to healthcare or when identifying poorly served areas through combinations of data relating to sociodemographic circumstances or supply/demand characteristics. However, different approaches are used. Guardiola (2004) has published complex measures of spatial accessibility to healthcare which can be classified into four categories [23]: provider-to-population ratios, distance to nearest provider, average distance to a set of providers and gravitational models of provider influence.

Apparicio *et al. *(2008) identified five commonly used measures of spatial accessibility: 1) the distance to closest service, 2) the number of services within a certain meters or minutes, 3) the mean distance to all services, 4) the mean distance to a certain number of closest services, and 5) the gravity model [5]. The gravity model assumes that the attractiveness of a service diminishes with distance and associated increasing travel impedance [14,24,25], while distance to the closest service or travel impedance is a simple and commonly used measure of spatial accessibility [26]. The main limitation of the distance to closest service method is that it only captures proximity between population and service locations with no account taken of availability [24].

Another method of studying spatial access to services based on population-to-provider ratios is the floating catchment area (FCA) method [27]. This method used circular buffers around census tract population centroids to compute a physician-to-population ratio from the number of enclosed facilities. It was argued that this 'floating catchment area' method allows cross-boundary flows by extending the buffer beyond the borders of the census tracts [14,28]. As with earlier versions of the gravity model, the FCA method was criticized for only taking into account supply, thus ignoring demand side of the equation [21,29]. It was only in the year 2000 that Radke and Mu [30] were able to address the supply-demand issue with the development of a spatial decomposition method which Luo and Wang popularized and referred to as the two-step floating catchment area (2SFCA) method [31,32].

There are some limitations with the 2SFCA method. One of these is its assumption that all services within the same catchment area are equally accessible by all residents. This is not always true since attractiveness of a clinic depends upon the number of physicians working in this clinic. The second main limitation is that previous studies using the 2SFCA method consider that all residents of a catchment area use services equally regardless of the population's characteristics. We argue that in light of recent demographic studies showing that health services use varies by age group [33], it is important to take these variations into account when modeling access to healthcare services.

Our study will overcome the above mentioned limitations by weighting the supply by the number of physicians available in each clinic and the demand by the percentage of persons who have used medical clinics during the year 2008-2009. To illustrate the significance of spatial access in the allocation of health care services, this paper applies GIS technology to evaluate the distribution of medical clinics in Montreal. The knowledge can be useful in helping the Ministry of health and social services to develop better systems for designating areas where medical clinics are lacking.

## Data and methods

The application of the 2SFCA needs three main parameters which are described in detail below: the supply, the demand and the computation of accessibility measures.

### The supply: Medical clinics

**Figure 1:**
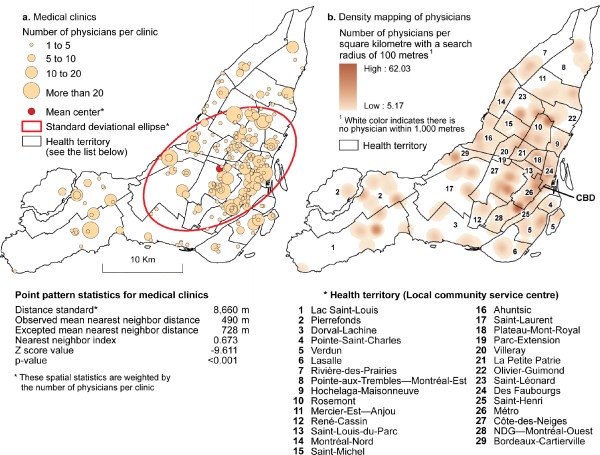
**Spatial distribution of medical clinics and physicians in Montreal (The supply)**.

A total of 1,344 physicians nested in 236 general medical clinics were integrated into a GIS software (ArcGIS version 9.3) and geo-coded using property parcels (Figure 1). Numerous authors suggested that the physician locations should be geo-coded by their street addresses [34]. However, recent studies reported that there are many potential problems with street geocoding and these problems may introduce bias and error in analyses [35-37]. In an recent article on the influence on geocoding quality on environmental exposure assessment of children living near high traffic roads [38], street geocoding was found to have a median error of 41 meters, a 90^th ^percentile of 100 meters, a 95^th ^percentile of 137 meters and a 99^th ^of 273 meters. In addition, street geocoding was found to consistently over-estimate the number of potentially exposed children at small distances up to 250 meters. Because we want to estimate accessibility at a small distance (500 m), it is very important to have a more accurate spatial location of medical clinics to avoid biases. That is why we have preferred to geocode medical clinics by residential property parcel.

Medical clinics and their location were inventoried from the website of the *Ministère de la santé et des services sociaux du Québec *(Québec Ministry of health and social services). This website currently provides the most accurate and comprehensive source of the location of medical clinics and the number of physicians in each clinic. To optimise the supply, medical clinics were weighted according to the number of physicians in each clinic. To well understand spatial distribution of medical clinics in the Montreal Island, we have 1) carried centrographic analysis, 2) calculated nearest neighbour index and 3) mapped density of medical clinics per DA (see appendix 1 for more explanation on different indices). Results of these analyses are reported in figure 1. It can be seen that medical clinics are clustered on the Island of Montreal (the nearest neighbour index is .673; p < .001). Moreover, the average distance between two neighbouring clinics is 490 meters.

### The demand: Potential medical clinics users

This study focuses on the Island of Montreal which is 483 km^2 ^and has a population of 1,906,811 inhabitants. Montreal is divided into 522 census tracts (CT) and 3,175 dissemination areas (DA). CT includes between 2,500 and 8,000 inhabitants versus 400 and 700 inhabitants for a DA [39]. Catchment area in this study refers to dissemination area, the smallest spatial unit used by Statistics Canada to provide census information. We exclude 28 DAs because they are uninhabited or without demographic data. The data used in this study are taken from the Statistics Canada census of 2006, the most recent census.

In the conventional method of the 2SFCA, the total population of each catchment area is used as the demand. But in this study, we weight the demand by the proportion of medical clinic users, in order to obtain a real world demand value. Data of the proportion of medical clinic users are from the Canadian Community Health Survey (CCHS) cycle 3.1 (2005-2006). The CCHS provides cross-sectional and longitudinal health and social data on a large sample of Canadian and is intended to provide reliable estimates of healthcare utilization at sub provincial scales of analyses. To obtain weighting coefficients, we grouped users in ten years groups (Table 1). With these proportions we then calculate for each DA, the potential users *W*_*k *_as:(1)

**Table 1:** Proportion of medical clinic users in Montreal in 2005-2006 (comparison with Quebec and Canada)

	Potential users		
Age group	Montreal	Quebec	Canada
0-14	.776	.570	.691
15-19	.606	.379	.564
20-29	.712	.439	.593
30-39	.729	.479	.639
40-49	.768	.544	.667
50-59	.814	.622	.732
60-69	.895	.711	.780
70-79	.861	.709	.804
80 +	.842	.645	.793

Source: Canadian Community Health Survey (CCHS) cycle 3.1

Where *P*_*x *_is the population of the group of the age group *x*. For instance, *P*_*0-14 *_is the population of the group 0 to 14 years.

Table 2 shows that the mean population per DA is about 586 persons with a weighted average of 453 potential medical clinic users.

**Table 2:** Descriptive statistics of population and potential users in dissemination areas on the Island of Montreal

Variable	Total population	Potential users
N	3,147	3,147
Mean	586	453
Std deviation	240	187

Minimum	113	96
Maximum	4,877	3,791
Percentiles		
5%	356	273
25% Q1	460	355
50% Median	542	417
75% Q3	648	501
95%	944	729

Source: Statistics Canada, Census of 2006.

### Measuring travel distance

One of the most important parameters in measuring the spatial accessibility is the distance between the supply and the demand locations. Various measures of distance ranging from Euclidian distance to travel times can be identified in the literature [4,5,40,41]. In their study on the accessibility to health services in Montreal, Apparicio *et al. *(2008) found a correlation of .992 between the shortest network distance and the shortest time distance [5]. Following this work, we selected the shortest network distance as our distance parameter (Travel-time should be used if distance is a poor measure of travel impedance e.g. if roads are unevenly distributed and travel speeds vary to great extent. For more detail on which impedance to use, see Wang, F. 2006). This parameter is computed using the CanMap Streetfiles from DMTI [42] and the Network Analyst extension of ArcGIS [43]. Four threshold travel distance values from the demand location were selected: 500 m, 1 Km, 2 Km and 3 Km.

## Methods

### Implementing the optimized two-step floating catchment area method

The conventional 2SFCA method computes the ratio of suppliers to residents within a service area centered at a supplier's location and sums up the ratios for residents living in areas where different suppliers' services overlap. This is quite innovative in comparison of the earlier versions of Floating Catchment Area Method which only considered supply location to compute the catchment area [14,24,25]. The 2SFCA uses either travel times or distance and is implemented in two steps [14,32]:

The first step assigns an initial ratio to each service area centered at a supply location as a measure of supply availability. For each supply location *j*, we search all demand locations *k *that are within a threshold Euclidian distance *d*_*0 *_from *j *and then compute the supply-to- demand ratio R_*j *_for 1,000 inhabitants within the catchment area [24]. The equation is:(2)

where d_*kj *_is the distance between *k *and *j*, D_*k *_is the demand at location *k *that falls within the catchment and finally S_*j *_is the capacity of supply (for example the number of medical clinics) at location *j *[14].

The second step consists of summing up the initial ratios in overlapped service areas to measure accessibility for a demand location, where residents have access to multiple supply locations [24]. For each demand location *i*, we search all supply locations *j *that are within the threshold distance *d*_*0 *_from location *i *and sum up the supply-to-demand ratios R*j *at those locations to obtain the full accessibility (F) A_*i*_^F ^at demand location *i*. The final equation of accessibility is:(3)

Where *d*_*ij *_is the distance between *i *and *j*, and *R*_*j *_the supply-to-demand ratio at supply location *j *that falls within the catchment centered at *i.*

But, since we want to correct for the number of physicians in each clinic and the proportion of medical clinic users for each catchment area, Equation 2 and Equation 3 will be optimized and become respectively:

and(4)

Where *P*_*j *_is the number of physicians in each medical clinic and *W*_*k *_the potential users of medical clinics during the past year per age group (see equation 1).

### Comparing the conventional and optimized 2SFCA methods: Statistical analysis

We then use different statistical techniques to compare the conventional and the optimized 2SFCA methods. These techniques include descriptive statistics, the Spearman's rank correlation and the t-test for independent samples [44]. The following step involves mapping out the differences between the two 2SFCA methods. To obtain these differences (D), we rescale the accessibility scores of the 2SFCA methods from 0 to 100 by subtracting the minimum from the score and dividing the result by the range as shown below:(5)

A negative value of D indicates that in comparison with the optimized method, the conventional method under-estimates accessibility and vice-versa. For instance, a value of -10 indicates that the conventional method under-estimates accessibility by 10% in comparison with the optimized method.

Finally, we calculate the classic Moran's *I *index [45,46] using a queen contiguity matrix to test if the differences were randomly distributed around the study catchment areas or autocorrelated. The following formula was used for the calculation:(6)

Where *n *is the number of spatial units indexed by *i *and *j*; *y *is the variable of interest;  is the mean of *y*; and *w*_*ij *_is the value of the queen contiguity matrix with value of 1 if *i *and *j *are adjacent or 0 if not.

## Results

### Mapping accessibility

**Figure 2:**
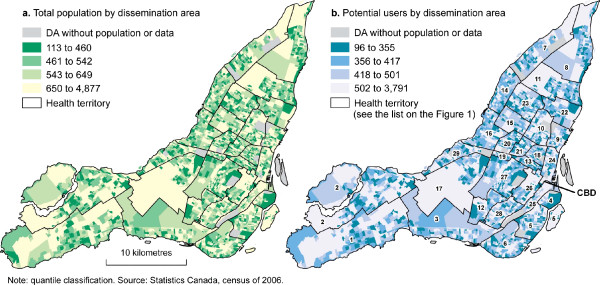
**Spatial distribution of population and potential users (the demand)**.

**Figure 3:**
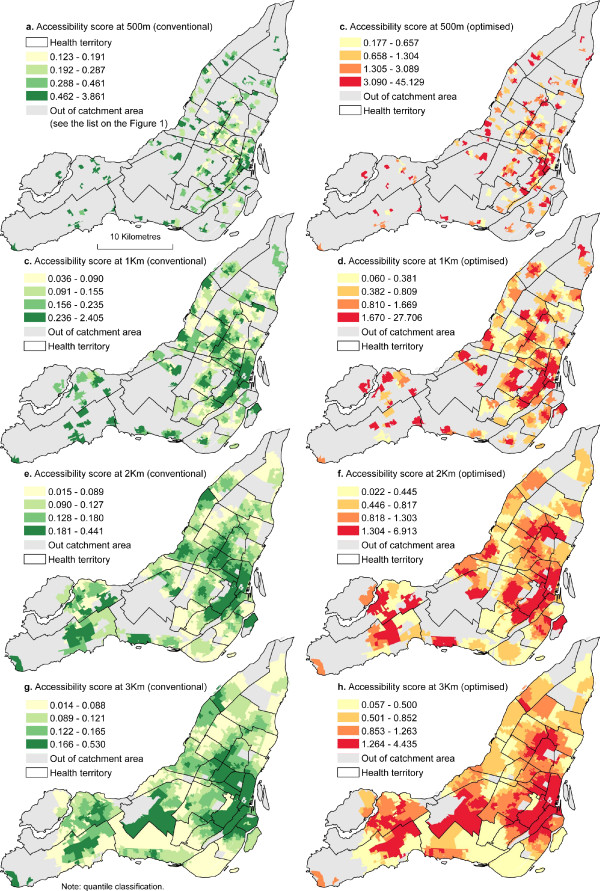
**Comparing accessibility scores of conventional and optimized 2SFCA method**.

By computing and evaluating the spatial accessibility index at 500 m, 1 Km, 2 Km and 3 Km shortest network distance, our results show that significant differences exist amongst DAs. Within a distance of 500 meters, the spatial accessibility score ranges from .123 to 3.861 with a standard deviation of .331 which means that within a distance of 500 meters, there is a minimum of .123 medical clinics accessible for 1,000 persons (figure 3). Accessibility scores calculated from the optimized method show greater variability than the conventional method (table 3). However, the optimized method increases more spatial accessibility score. Within a distance of 1 Km, for instance, the maximum clinic accessible for 1,000 persons in one DA, when it is estimated by the conventional method is approximately 2.404, compared with 27.706 physicians for 1,000 potential users when it is estimated by the optimized method. Figure 3 also shows that whatever the method, regions outside the downtown area generally have a much lower spatial accessibility score and this difference tends to increase with the distance. The mean value of the spatial accessibility at 3 Km travel distance is 0.131 for the conventional method and 0.97 for the optimized method. However, considering the very high spatial accessibility score, the size of the population residing in the CBD (Central Business District) area as shown on figure 2, the overall spatial accessibility score of the whole island at a 3 Km level is quite low. The conventional method indicates that the number of medical clinics available for 1,000 persons varies from .014 to .530 and the optimized methods shows that there are between .057 and 4.435 physicians for 1,000 potential users available at 3 Km.

**Table 3:** Descriptive statistics of accessibility scores

	Conventional 2SFCA method				Optimized 2SFCA method			
Bandwidth	500 m	1 Km	2 Km	3 Km	500 m	1 Km	2 Km	3 Km
N	1002	2118	2886	3072	1002	2118	2886	3072
Mean	0.3857	0.1859	0.1391	0.1309	2.7904	1.3819	1.0315	0.9685
Std. Dev.	0.3313	0.1409	0.0744	0.0598	4.4716	2.0370	0.8484	0.6239
Min	0.1231	0.0356	0.0145	0.0141	0.1771	0.0597	0.0216	0.0575
Max	3.8610	2.4047	0.4408	0.5304	45.1294	27.7059	6.9133	4.4355
Percentiles								
5%	0.1352	0.0566	0.0388	0.0512	0.2488	0.1027	0.1652	0.2851
25%	0.1912	0.0904	0.0892	0.0879	0.6571	0.3813	0.4454	0.4996
50%	0.2866	0.1549	0.1266	0.1214	1.3038	0.8091	0.8174	0.8497
75%	0.4570	0.2331	0.1797	0.1646	3.0537	1.6693	1.2974	1.2627
95%	0.9921	0.4137	0.2860	0.2474	9.0165	4.0747	2.8478	2.1555

This indicates that most residents will need to travel more than 3 Km to have access to a medical clinic. This is particularly the case for the west, the central and the north parts of the island that have the lowest level of poor accessibility to medical clinics.

### Comparison of the conventional and the optimized 2SFCA methods

The global analysis of differences is performed by Spearman's rank correlation between the four thresholds distances used to calculated accessibility in this study. Results are shown in table 4. It can be seen that correlation values increase with distance indicating that when we use the conventional 2SFCA method, potential errors decrease with distance. Concretely, conventional and optimized methods tend to have the same estimation at highest distances.

**Table 4:** Spearman's rank correlation coefficients between accessibility score of conventional and optimized 2SFCA methods

		Optimized 2SFCA method			
		500 m	1 Km	2 Km	3 Km
Conventional	500 m	.595**			
2SFCA	1 Km		.729**		
method	2 Km			.760**	
	3 Km				.786**

** p < .0001

**Figure 4:**
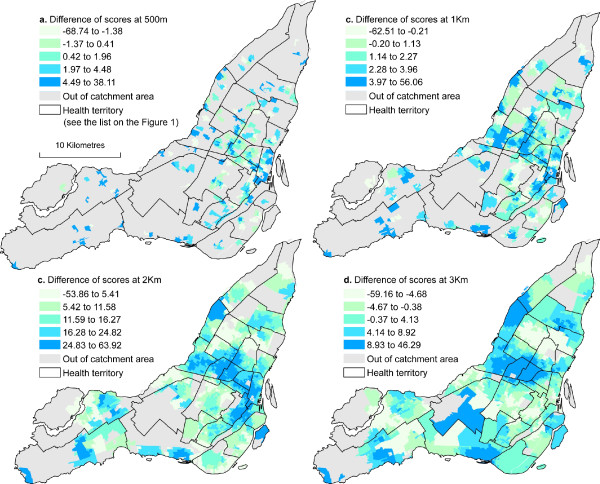
**Spatial distribution of differences between conventional and optimized 2SFCA methods**.

For recall, to obtain results of figure 4, we first calculated differences between the conventional and the optimized 2SFCA method rescaled from 0 to 100 (see equation 5 in the method's section). Results presented in table 5 confirm the assertion that great differences between the conventional and the optimized methods mostly occurred at the lowest distance and these differences are all significant. Globally, compare to the optimized method, the conventional 2SFCA method over-estimates accessibility. At 500 m for instance, the over-estimation is greater than 7.53% and 10.55% for respectively 10% and 5% of cases (see percentiles 90% and 95% in table 5). However, the errors are highest at 3 km with an under-estimation of 9.62% and more for 5% of cases and over-estimation of 14.22% and more for 5% of cases (see percentiles 5% and 95% in table 5).

**Table 5:** Differences between the conventional and the optimized 2SFCA methods

Bandwidth		t-test	Percentiles						
	Mean	p-value	5%	10%	25%	50%	75%	90%	95%
500 m	1.21	0.004	-8.25	-4.31	-1.03	1.02	3.74	7.53	10.55
1 Km	1.56	<.0001	-4.05	-2.06	0.09	1.68	3.45	5.42	7.55
2 Km	14.56	<.0001	-1.97	2.12	6.65	13.85	22.25	30.09	33.95
3 Km	1.82	<.0001	-9.62	-6.98	-3.49	2.45	7.20	12.51	14.22

Figure 4 shows the spatial distribution of the errors previously mentioned. For clarification, a positive score in figure 4 indicates an over-estimation of accessibility by the conventional method. The highest error scores are detected in the west of the island. But in general, as distance increases, the conventional 2SFCA method tends to over-estimate accessibility in the CBD and its surrounding. Figure 4 a shows few errors because many DAs at this distance are out of the catchment areas.

Table 6 presents the results of the autocorrelation index of the differences. It can be noted that as the distance increases, differences are more autocorrelated, indicating that differences between the optimized and the conventional 2SFCA methods are only concentrated in some areas of the Island.

**Table 6:** Spatial autocorrelation of differences between the conventional and the optimized 2SFCA methods

	Queen contiguity matrix				Inverse distance squared matrix			
	500 m	1 Km	2 Km	3 Km	500 m	1 Km	2 Km	3 Km
Moran's Index	0.456	0.596	0.847	0.844	0.259	0.394	0.842	0.707
Z Score	19.017	41.730	71.630	75.087	14.319	29.831	74.018	72.621
p-value	0.000	0.000	0.000	0.000	0.000	0.000	0.000	0.000

## Discussion

In this study, medical clinics were found to be clustered and concentrated in the central part of the Island. This is not surprising given the attractiveness of the city center for economic activities. Our results concord with those reported in previous studies [47-49]. How may this location place be explained particularly in the Canadian context where healthcare is free of charge for all citizens? In fact, even if all Canadians are covered by public health insurance, the number of patients is taken into account for the funding of each medical clinic and given the concentration of population in the central part of Montreal, physicians may choose these places to benefit from a higher density of potential clients. Thus, the concept of a trade area may be a possible explanation for the concentration of medical clinics near the city center. A trade area can simply be defined as the geographic area from which a business draws most of its customers.

Another finding is that at first glance, spatial accessibility to medical clinics in Montreal is reasonably good and is well estimated by the conventional 2SFCA method. We realised that the optimized method ameliorated significantly accessibility scores. At 3 Km for instance, accessibility scores range from 0.014 to .530 clinics per 1,000 inhabitants for the conventional model and from .057 to 4.435 physicians per 1,000 potential clients for the optimized model.

Results of the comparison between conventional and optimized 2SFCA methods indicate that optimized method effectively estimates accessibility at low distance (500 meters or less). However, even if there is no great differences between the two approaches at highest distances, conventional method tends to over-estimate accessibility scores. Also, the positive autocorrelation of measurement errors increases with the distance. To our knowledge, this is the first study using an optimized 2SFCA method. Further studies are required to confirm our findings, in particular in rural areas by using the shortest distance time such as the study of McGrail and Humphreys [24].

Our results are a valuable contribution to existing scientific literature in the field of spatial accessibility to healthcare. By determining areas that are outside of the catchments, we can identify regions for which health system administrators should consider the spatial location of further medical clinics in Montreal. Figure 2 highlights the urban areas in Montreal that are without spatial access to medical clinics, specifically those located greater than 3 Km traveling distance.

Our analysis has some limitations. One of these limitations is the aggregation-error. Aggregation-error arises when measuring distance from aggregated areal units to facilities, and results from using a single point as a proxy for the locations of individuals within the area units [50]. We have attempted to reduce aggregation error by integrating the dissemination blocks data since they better reflect the spatial distribution of individuals [37,50].

As with other studies in spatial analysis, our study deals with difficulties arising from the ecological fallacy and the modifiable areal unit problem (MAUP). Ecological fallacy generally occurs when a researcher makes an inference about an individual based on aggregate data for a group [51]. We have not made such inferences. Sample size was not a problem for us because we used the entire population of Montreal and were able to geocode all medical clinics. The Modifiable Areal Unit Problem (MAUP) is a potential source of error that can affect spatial studies which utilise aggregate data sources. «The essence of the MAUP is that there are many ways to draw boundaries to demarcate space into discrete units to form multiple spatial partitioning systems » [52]. The problem which arises is that MAUP can introduce biases in spatial analysis results if the areal unit is not well defined. To avoid this biases, we have used the DAs as our unit of analysis because they are an objective measure of neighbourhood [53]. Like any accessibility study, results near the borders of Montreal need to be interpreted with caution because of edge effects. In other words, medical clinics outside of Montreal should also contribute to accessibility of residents near the borders, but are not accounted for in this study.

It is worth noting that, as in many other studies on spatial accessibility, our method concerns only potential spatial accessibility, not revealed access (actual utilization of medical clinics). Only complex and expensive investigations can reveal the absolute significance of spatial accessibility for utilization, or the relative importance of spatial accessibility toward the other components of medical clinics access. Also note that only spatial accessibility is considered in this study. It is possible that residents in areas with high scores of spatial accessibility (as in the inner city) may not actually enjoy good access to medical clinics thus aspatial factors also play important roles in affecting accessibility.

Despite these limitations, our findings underline a number of the strengths of this study. The first strength is the methodological contribution of our study. It is clear that optimization of the 2SFCA method enable us to reveal areas with physician shortage at small distances. Also, the capabilities of geographic information systems (GIS) to handle large amounts of data over large geographic areas at fine levels of geographic detail makes them ideally suited to measure geographical accessibility to medical clinics and other healthcare services. The use of GIS facilitates the production of geographical accessibility measures that overcome the limitations of traditional statistics based on service to population ratio and Euclidian distances.

## Conclusions

Access to health care services will continue to be among the most important preoccupations in developed countries during the next decades. It is then important to develop and implement methodological methods of analysis to determine areas of healthcare services shortage that will aide in planning the locations of new health care services. The 2SFCA method enables the calculation of spatial accessibility at a much finer spatial resolution compared to population-to-provider ratios, thus significantly advancing the measurement of spatial accessibility. Our research has demonstrated the application of the conventional and an alternative method of the 2SFCA method for the Island of Montreal, using medical clinics as the health service of interest. Results have revealed only minor variations in the pattern of spatial accessibility between the conventional and the optimized 2SFCA methods. However, closer examination of results demonstrates that the optimized 2SFCA method is the best estimator of accessibility when the distance is not larger than 500 m. Nevertheless, future studies investigating the contribution of the optimized 2SFCA method in rural contexts and also in other cities are needed to conclude whether this method ameliorates or not the measure of spatial accessibility.

Despites these reservations, the study represents an important step in understanding the integration of GIS approaches to health services accessibility. The research methodology developed in this paper is also useful for health planners and other researchers in the field of public health. These researches usually use the GIS to generate contextual variables for both socioeconomic and physical environment.

## Competing interests

The authors declare that they have no competing interests.

## Authors' contributions

ANN and PA are the principal investigators of the study. They carried out the GIS, statistical and mapping analyses. ANN reviewed the literature and wrote parts of the paper. All authors jointly drafted and critically revised the paper, and read and approved the final manuscript.

## Appendix 1

The **mean center **is defined as the location of a single x,y coordinate value that represents the average x-coordinate value and the average y-coordinate value of all features in a study area [54]. For simplicity, we will define it as the central or the average location of a set of points [55] (which in this study represents the medical clinics in Montreal). We have selected the weighted mean center to take into account the number of physicians per medical clinic. We have used the following formula:

Where  and  are the coordinates of the weighted mean center, *x*_*i *_and *y*_*i *_are the coordinates of point *i*, *n *is the number of points, and *w*_*i *_is the weight at point *i.*

The **standard deviational ellipse **measures whether a distribution of features exhibits a directional trend (whether features are farther from a specified point in one direction than in another direction). A high standard deviation value indicates greater dispersion of the features around the center. For the weighted standard deviation, we have used the following:

The **nearest neighbour index **measures the degree of spatial dispersion in the distribution based on the minimum of the inter-feature distances [56]. The magnitude of this standard error indicates how likely any difference between the observed average nearest neighbour distance and the expected pattern is to occur purely by chance. The nearest neighbour index was obtained by the following equation:

Where *d*_*i *_is the distance between point *i *and its nearest neighbours; *n *the number of points and *A *the area of the referenced zone.

## Pre-publication history

The pre-publication history for this paper can be accessed here:

http://www.biomedcentral.com/1472-6963/11/166/prepub
